# Herpesvirus Glycoproteins Undergo Multiple Antigenic Changes before Membrane Fusion

**DOI:** 10.1371/journal.pone.0030152

**Published:** 2012-01-09

**Authors:** Daniel L. Glauser, Anne-Sophie Kratz, Philip G. Stevenson

**Affiliations:** Division of Virology, Department of Pathology, University of Cambridge, Cambridge, United Kingdom; University of Pittsburgh School of Medicine, United States of America

## Abstract

Herpesvirus entry is a complicated process involving multiple virion glycoproteins and culminating in membrane fusion. Glycoprotein conformation changes are likely to play key roles. Studies of recombinant glycoproteins have revealed some structural features of the virion fusion machinery. However, how the virion glycoproteins change during infection remains unclear. Here using conformation-specific monoclonal antibodies we show *in situ* that each component of the Murid Herpesvirus-4 (MuHV-4) entry machinery—gB, gH/gL and gp150—changes in antigenicity before tegument protein release begins. Further changes then occurred upon actual membrane fusion. Thus virions revealed their final fusogenic form only in late endosomes. The substantial antigenic differences between this form and that of extracellular virions suggested that antibodies have only a limited opportunity to block virion membrane fusion.

## Introduction

Enveloped viruses penetrate cells by membrane fusion. Herpesvirus fusion requires gB and gH, which forms a heterodimer with gL [1]. A directly fusogenic role for gB is implied by its structural homology to the Vesicular stomatitis virus glycoprotein G (VSV-G) [2], [3]. gH/gL does not appear to be a typical fusogen [4], but has a conserved hydrophobic patch that could participate in fusion [5]. An additional virion glycoprotein often regulates gH/gL/gB. For example the Herpes simplex virus (HSV) gD brings together gH/gL and gB in transfected cells after receptor binding [6]–[8], although how this works is still debated.

Membrane fusion is driven by conformation changes in virion glycoproteins [9], [10]. The rationale for pre-fusion glycoprotein changes such as that of the HSV gD [6] is less clear. One possibility is antibody evasion, and a post-binding conformation change in the HIV gp120 has been proposed to limit the exposure to antibody of its functionally important epitopes [11]. However gD remains a prominent neutralization target for HSV despite its change [12], and although low pH induces changes in the post-fusion HSV gB [13] pre-fusion changes have not been identified in either gB or gH/gL.

A full understanding of herpesvirus membrane fusion may require analysis beyond HSV. Murid Herpesvirus-4 (MuHV-4) provides an accessible way to study gamma-herpesvirus infection [14], [15]. Like other mammalian herpesviruses, it encodes homologs of gH and gB that are essential for infectivity [16]. MuHV-4 infects epithelial cells by first binding to heparan sulfate (HS) via gp70 [17] or gH/gL [18]. Virions are then endocytosed and show pH-dependent capsid release from late endosomes [19]. Conformation-dependent monoclonal antibodies (mAbs) have identified antigenic changes in gB and gH that broadly coincide with membrane fusion. Thus extracellular virion gB (by definition pre-fusion) is recognized by mAb BN-1A7 but not by mAb MG-1A12 (BN-1A7^+^MG-1A12^-^), whereas after capsid release from late endosomes gB (now by definition post-fusion) is BN-1A7^-^MG-1A12^+^ [20]. Similarly while extracellular virions have gH mostly bound to gL [21], late endosomal virions lose gL-dependent epitopes (gH/gL) and retain gL-independent epitopes (gH-only), implying gH/gL dissociation or an analogous conformation change [22]. However, whether these changes are part of the fusion reaction or simply associated with it has been unclear.

Not all the gH and gB on herpes virions need necessarily be engaged for membrane fusion to occur. Nevertheless the engagement of one glycoprotein complex presumably lowers the threshold for subsequent engagements, and the fraction of the available glycoproteins on enveloped virions that engage in fusion seems to be high [10]. Thus the fusogenic role of gB and the coincidence of its antigenic switch with capsid release argue that this reflects a fusion-associated conformation change. The situation for gH is less clear: a constitutive association with gB [23] would suggest that it undergoes inter-dependent conformation changes, but gL is non-essential for infection [24], implying that gH/gL epitope loss is not intrinsic to the fusion reaction.

Gp150 is another component of the MuHV-4 entry complex [23]. Like gL it is non-essential for infectivity; indeed gp150^-^ virions are more infectious than the wild-type, in that they are less HS-dependent [25], [26]. Thus gp150 regulates HS-independent cell binding. The gp150 homologs of EBV (gp350) and BoHV-4 (gp180) also have regulatory functions: gp180^-^ BoHV-4 shows enhanced infection of HS-deficient cells [27], and gp350^-^ EBV shows enhanced infection of epithelial cells [28]. Each of these glycoproteins is presumably displaced during wild-type virion entry to relieve its inhibitory effect. However corresponding antigenic changes have not been identified. Here we show that the MuHV-4 gB, gH and gp150 all undergo antigenic changes upstream of membrane fusion, with different blocks to membrane fusion arresting virions in different intermediate states. Thus the entry complex engaging in fusion was substantially different to that displayed on extracellular virions. Since MuHV-4 infection is endocytic this may substantially protect the fusogenic form against extracellular antibodies.

## Results

### Two distinct antigenic changes in gB

**Figure 1:**
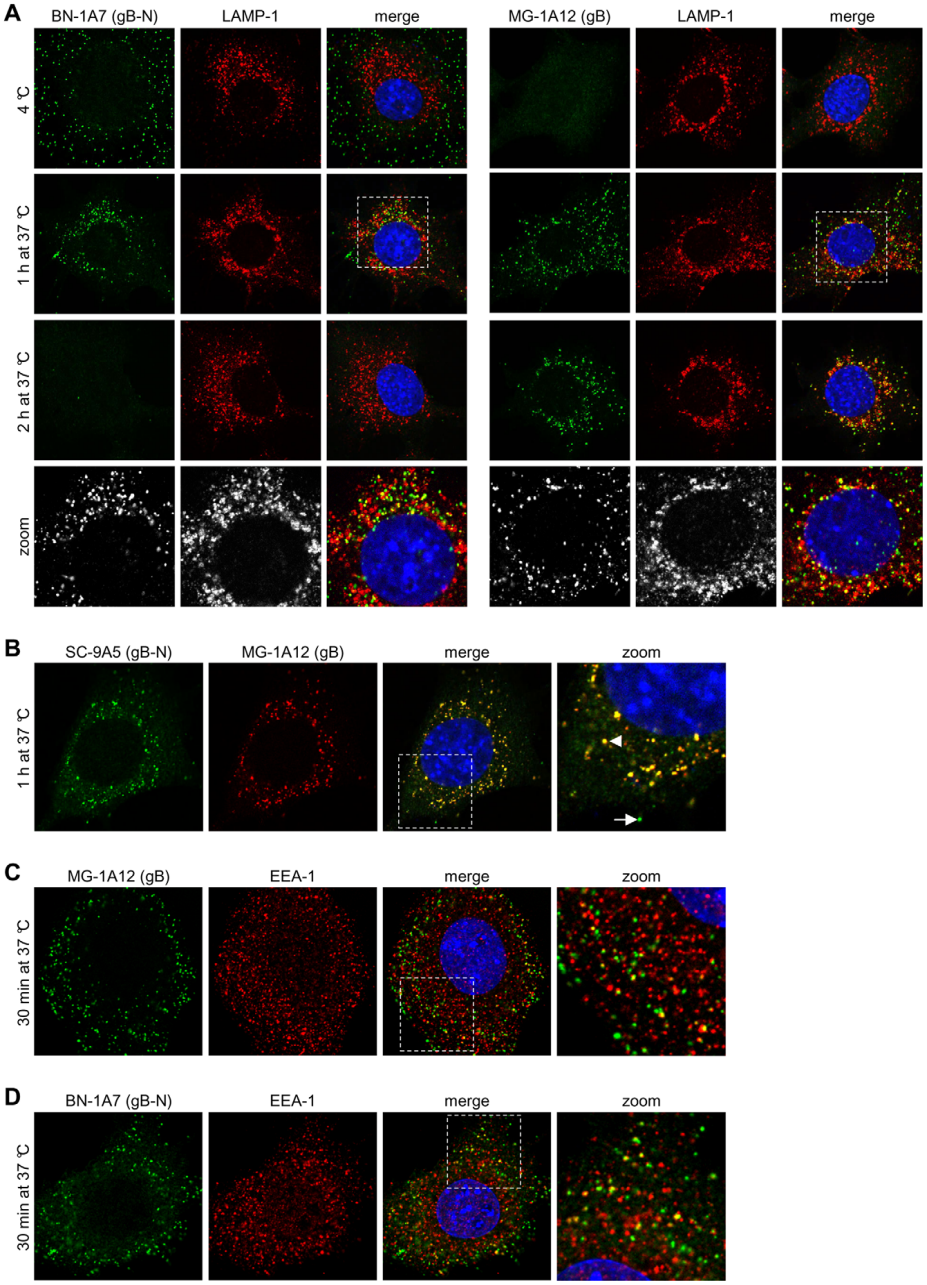
Kinetic analysis of antigenic changes in gB. **(A)** NMuMG cells were incubated with MuHV-4 (3 p.f.u./cell, 2h, 4°C), washed, and then either fixed immediately or first further incubated (1h and 2h, 37°C) to allow virion endocytosis. The cells were then stained with the gB-specific mAbs BN-1A7 and MG-1A12 (green). BN-1A7 (IgG_2a_) recognizes only pre-fusion gB; MG-1A12 (IgG_2a_) recognizes a gB epitope that is inaccessible on extracellular virions but becomes accessible after endocytosis. The cells were also stained for LAMP-1 (red) and counter-stained with DAPI (blue). **(B)** NMuMG cells were incubated with MuHV-4 (3 p.f.u./cell, 2h, 4°C), washed, and then either fixed immediately or first further incubated (1h, 37°C) to allow virion endocytosis. The cells were then co-stained with the gB-specific mAbs SC-9A5 (IgG_3_, green) and MG-1A12 (IgG_2a_, red). SC-9A5 recognizes only pre-fusion gB. The arrow points to a gB focus in the extracellular SC-9A5^+^MG-1A12^-^ antigenic state; the arrowhead points to a focus of gB that is both SC-9A5^+^ and MG-1A12^+^. **(C)** NMuMG cells were incubated with MuHV-4 (3 p.f.u./cell, 2h, 4°C), washed, and fixed after a further incubation to allow virion endocytosis (30 min, 37°C). The cells were then stained with the gB-specific IgG_2a_ MG-1A12 (green) and for EEA-1 (red), and counter-stained with DAPI (blue). **(D)** The cells were infected and processed as in **(C)** but stained with the gB-specific IgG_2a_ BN-1A7 (green). In this and all subsequent figures, the data shown are representative of at least 100 cells examined. Note that MuHV-4 plaque titers underestimate virion numbers 10-100 fold.

The glycoprotein conformations of cell-bound, non-endocytosed virions must be pre-fusion; after capsid release from late endosomes they must at least in part be post-fusion. Between cell binding and capsid release MuHV-4 virions lose gH/gL epitopes, while gB switches from BN-1A7^+^MG-1A12^-^ to BN-1A7^-^MG-1A12^+^. To define better the relationship between these antigenic switches, we undertook a kinetic analysis of virion entry, binding virions to cells at 4°C, then incubating them for different times at 37°C to allow endocytosis and observing how their antigenicity changed (Fig. 1).

We first analysed gB (Fig. 1A). After cell binding at 4°C, virions were detected by mAb BN-1A7 but not by mAb MG-1A12. After a 1h 37°C incubation, virions were detected by both mAbs in late endosomes, based on co-localization with LAMP-1. After 2h at 37°C, virions were detected with mAb MG-1A12 but not with BN-1A7. Therefore MG-1A12 epitope gain preceded BN-1A7 epitope loss.

Endocytic MuHV-4 transport is not completely synchronous, and glycoprotein antigenicity correlates principally with virion site - whether inside or outside late endosomes [20], [22]. Thus at the intermediate 1h timepoint the BN-1A7^+^ and MG-1A12^+^ virions were possibly distinct populations at different stages of infection. To compare them more directly we used mAb SC-9A5, which like BN-1A7 recognizes gB only before capsid release [29] but is IgG_3_ and so allows isotype-specific co-staining with MG-1A12 (IgG_2a_). After 1h at 37°C. SC-9A5^+^ and MG-1A12^+^ virions co-localized in individual endosomes (Fig. 1B). Therefore virions in the same intracellular site expressed a gB epitope present post- but not pre-fusion (MG-1A12), and one present pre- but not post-fusion (SC-9A5). The co-existence of these epitopes suggested that gB might have an additional intermediate state that was neither stably pre-fusion nor post-fusion.

MG-1A12 staining also appeared very early (30 min) after switching cell-bound virions to 37°C (Fig. 1C). At this time it overlapped in localization with the early endosome marker EEA-1, as did BN-1A7 staining (Fig. 1D). Thus virions adopted the BN-1A7^+^MG-1A12^+^ intermediate state before they reached late endosomes.

### gH also shows distinct antigenic changes

**Figure 2:**
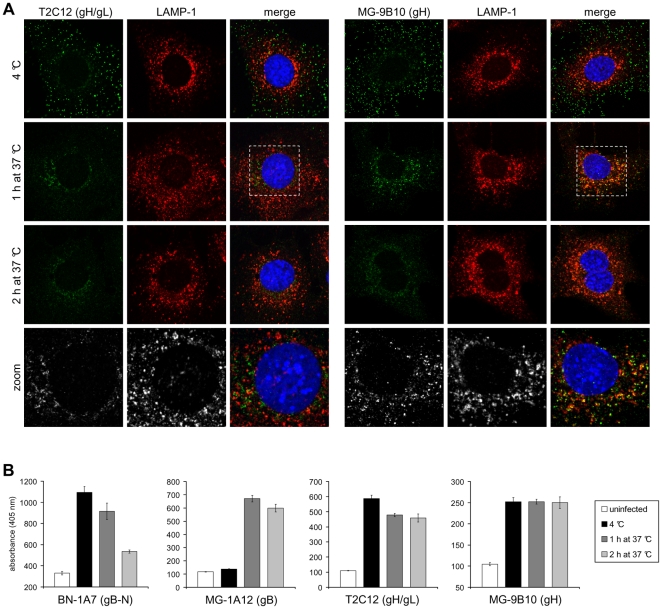
Kinetic analysis of antigenic changes in gH. **(A)** NMuMG cells were incubated with MuHV-4 (3 p.f.u./cell, 2h, 4°C), washed, and then either fixed immediately or first further incubated (1h and 2h, 37°C) to allow virion endocytosis. The cells were then stained with the gH-specific mAbs T2C12 and MG-9B10 (green). T2C12 (IgG_2a_) recognizes only gH complexed with gL (gH/gL); MG-9B10 (IgG_2a_) recognizes only gH which is not associated with gL (gH-only). The cells were also stained for LAMP-1 (red) and counter-stained with DAPI (blue). **(B)** Cells were infected and processed as in **(A)**. Infected cells and uninfected control cells were then incubated with the gB-specific mAbs BN-1A7 (IgG_2a_) and MG-1A12 (IgG_2a_), the gH/gL-specific mAb T2C12 (IgG_2a_) and the gH-only-specific mAb MG-9B10 (IgG_2a_). Bound antibody was detected with an alkaline phosphatase-conjugated secondary antibody and incubation with p-nitrophenyl phosphate substrate. The bars show mean ± SEM values from 6 wells. The experiment shown is representative of two equivalent experiments.

After 2h at 4°C, cell-bound virions were detected by both mAb T2C12 (gH/gL) and mAb MG-9B10 (gH-only), but after a further 1h at 37°C these mAbs showed distinct staining patterns (Fig. 2A). gH-only staining, like that of gB showed co-localization with LAMP-1; in contrast, gH/gL staining was reduced and the residual staining was peri-nuclear with little or no LAMP-1 co-localization. After 2h at 37°C neither mAb co-localized with LAMP-1. Thus again virions appeared to pass through two distinct stages: loss/redistribution of gH/gL, then loss/redistribution of gH-only.

### Virion antigenic changes measured by ELISA

We used ELISA to quantitate the antigenic changes in gB and gH/gL across cell populations (Fig. 2B). Infection conditions were the same as for immunofluorescence, but after cell fixation and permeabilization we detected antibody binding with an alkaline phosphatase-conjugated secondary antibody and a soluble, colorimetric substrate. Fig. S1 shows corresponding immunofluorescence images. The different assay results correlated well for gB: first the MG-1A12 epitope appeared, then the BN-1A7 epitope was lost. However the gH-only signal remained surprisingly constant after 2h at 37°C. Thus gH-only appeared to be more redistributed from late endosomes than lost. gH/gL detection by mAb T2C12 was reduced by incubation at 37°C but less markedly than that of gB detection by mAb BN-1A7. Again this would be consistent with the lack of late endosomal gH/gL being partly due to redistribution. A similar lack of late endosomal gH/gL by immunofluorescence and partial loss by ELISA was seen with mAb 7E5, which recognizes a distinct gH/gL epitope [21] (Fig. S2).

### High endosomal pH and gB-directed neutralization arrest the virion fusion complex in different intermediate states

**Figure 3:**
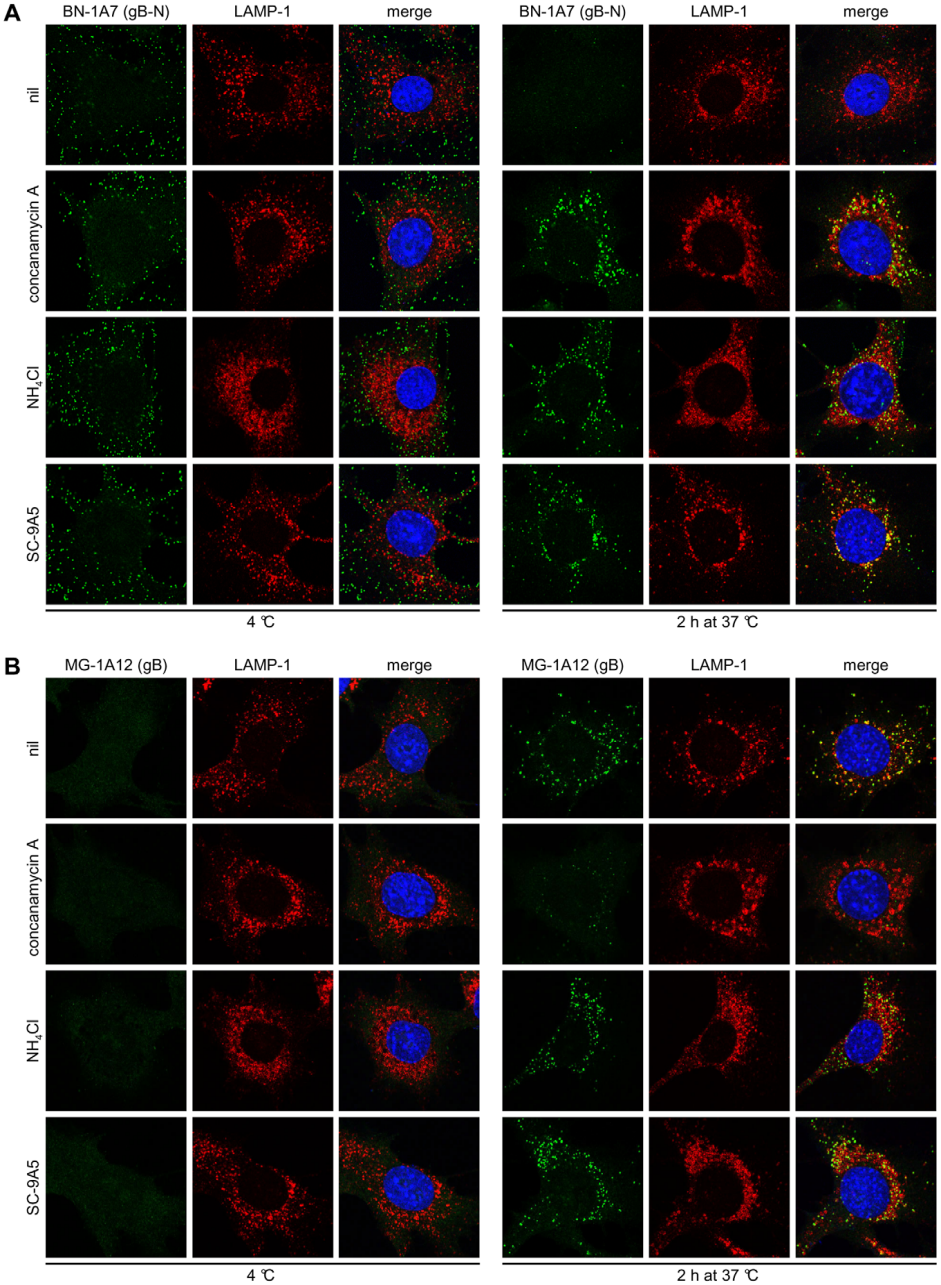
Concanamycin A and NH_4_Cl treatments and virus neutralization by mAb SC-9A5 arrest gB in distinct antigenic states. MuHV-4 (3 p.f.u./cell) was left untreated (nil) or pre-incubated (2h, 37°C) with 400 µg/ml SC-9A5 (IgG_3_) before binding to NMuMG cells (2h, 4°C). For drug treatments, cells were incubated with 1 µM concanamycin A and 25 mM NH_4_Cl before adding virus (2h, 37°C) and during binding (2h, 4°C). Unbound virions were then removed by washing and the cells either fixed immediately or after a further incubation (2h, 37°C) in presence or absence of antibodies and drugs. In **(A)** the cells were stained with the gB-specific IgG_2a_ BN-1A7 and in **(B)** with the gB-specific IgG_2a_ MG-1A12 (green). The cells were also stained for LAMP-1 (red) and with DAPI (blue). Treating cells with DMSO alone had no effect (not shown). Treatment of cells with concanamycin A arrests gB in its extracellular BN-1A7^+^MG-1A12^-^ form, while treatment with NH_4_Cl and virus neutralization by SC-9A5 arrest gB at an intermediate BN-1A7^+^MG-1A12^+^ state.

Raising the endosomal pH of virus-exposed cells with concanamycin A (ConA) prevents MuHV-4 infection and keeps gB in its pre-fusion state - BN-1A7^+^MG-1A12^-^ [20], [22] (Fig. 3). In contrast, raising the endosomal pH with ammonium chloride (NH_4_Cl) blocked BN-1A7 epitope loss without preventing MG-1A12 epitope display, as did blocking membrane fusion with the gB-specific neutralizing mAb SC-9A5 [29] (Fig. 3). None of these treatments affected endocytic virion transport or staining by a pan-gB-specific mAb, MG-4D11 (Fig. S3). Thus NH_4_Cl and SC-9A5 treatments stably arrested gB in LAMP-1^+^ late endosomes in its intermediate BN-1A7^+^MG-1A12^+^ state.

**Figure 4:**
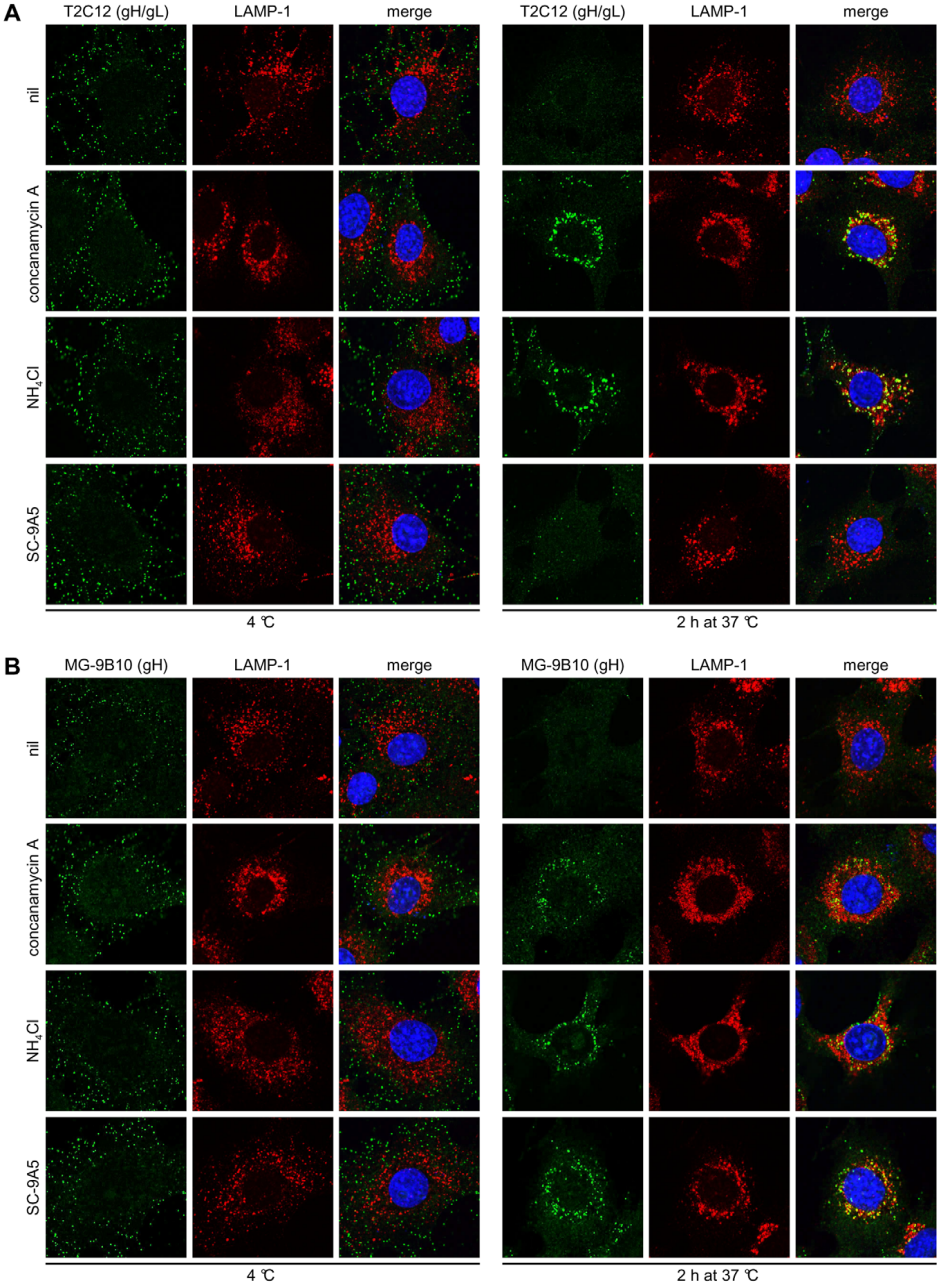
gH undergoes pre-fusion and fusion-related antigenic changes. Infections, drug treatments and antibody treatments were as for Figure 3. In **(A)** the cells were stained with the gH/gL-specific IgG_2a_ T2C12 and in **(B)** with the gH-only-specific IgG_2a_ MG-9B10 (green). The cells were also stained for LAMP-1 (red) and with DAPI (blue). Treating cells with DMSO alone had no effect (not shown).

**Figure 5:**
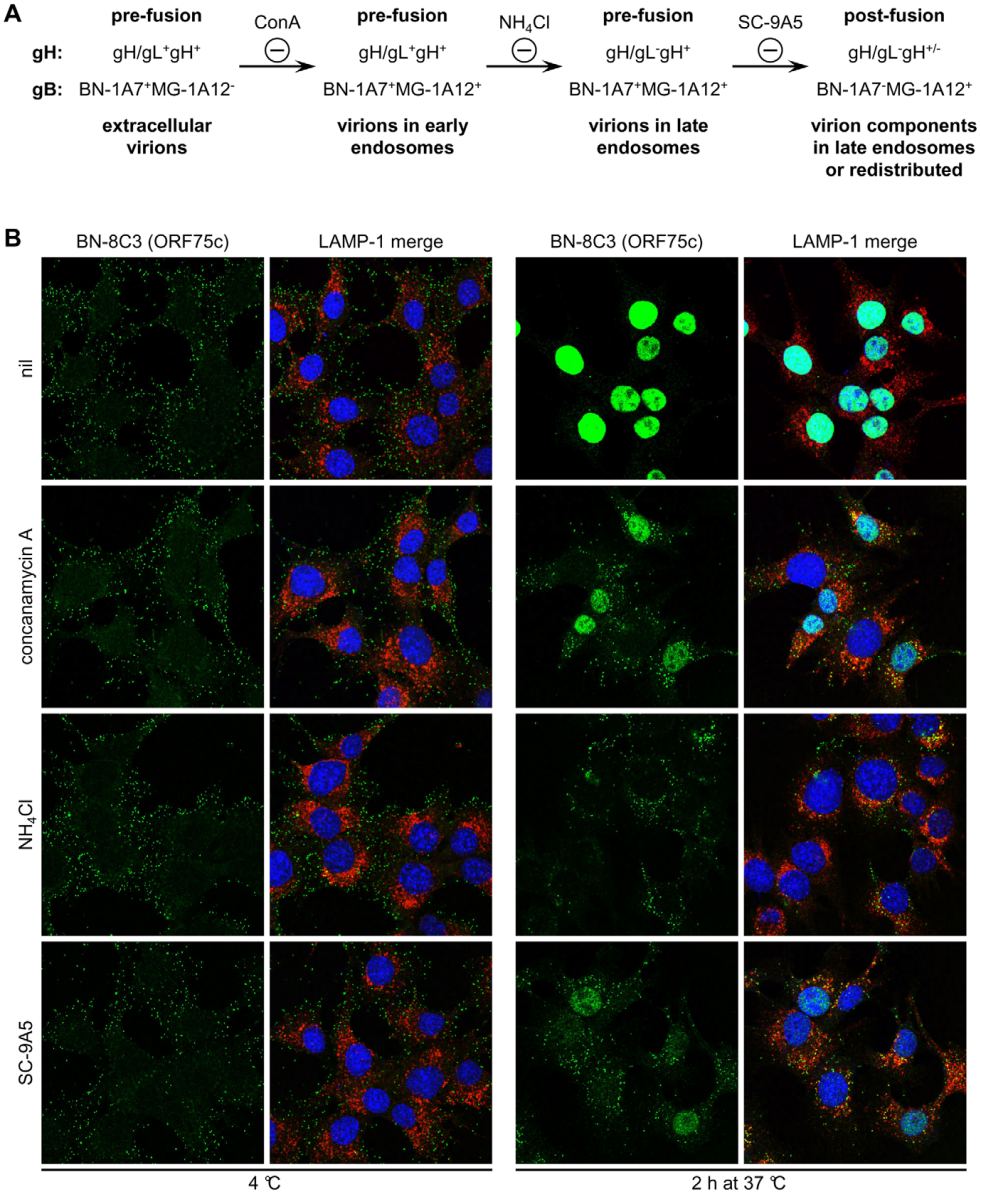
gH and gB antigenic changes during virus entry and the effect of drug treatments and antibody-mediated neutralization on ORF75c tegument protein release. (A) A schematic diagram summarizes the progress of antigenic changes in gH and gB during virion entry, and the distinct blocks to this progresses effected by concanamycin A (ConA), NH_4_Cl, and antibody-mediated neutralization by SC-9A5. gH/gL epitopes are lost upstream of fusion, while gH-only epitopes are lost/redistributed upon membrane fusion. The MG-1A12 gB epitope is revealed upstream of fusion, while the BN-1A7 epitope disappears upon fusion. Both NH_4_Cl treatment and virus neutralization by mAb SC-9A5 arrest gB in an intermediate BN-1A7^+^MG-1A12^+^ state. (B) Infections, drug treatments and antibody treatments were as for Figure 3. The cells were stained with the ORF75c-specific IgG_1_ BN-8C3 (green), a LAMP-1-specific mAb (red), and DAPI (blue). All images were taken with the same confocal settings. Incubating cells with DMSO alone had no effect (not shown). Concanamycin A and NH_4_Cl treatments and virus neutralization by SC-9A5 all inhibit viral membrane fusion and ORF75c tegument protein release.

We examined the same inhibitors for their effect on gH (Fig. 4). Again virions were bound to cells at 4°C, then incubated at 37°C to allow endocytosis. ConA and NH_4_Cl treatments blocked gH/gL epitope loss/redistribution, while mAb SC-9A5 did not (Fig. 4A). All 3 treatments blocked gH-only epitope loss/redistribution (Fig. 4B). Thus ConA kept virions in their extracellular form (gH/gL^+^BN-1A7^+^MG-1A12^-^); NH_4_Cl preserved gH/gL in late endosomes but kept gB as BN-1A7^+^MG-1A12^+^; and SC-9A5 had the same effect on gB but arrested gH as gH-only rather than as gH/gL. Fig. 5A summarizes these 3 different blocks.

Figure 5B shows how much each block inhibited tegument protein release. The abundant tegument protein encoded by ORF75c is rapidly transported to the cell nucleus after release [30] and so provides a useful marker of virion membrane fusion. ORF75c staining also shows a marked increase in intensity after fusion, presumably because protein confined within the virion tegument is poorly accessible to antibody [29]. NH_4_Cl and SC-9A5 treatments blocked ORF75c release at least as effectively as did ConA (Fig. 5B), and each was effective for at least 6h (Fig. S4). Therefore the entry blocks by ConA, NH_4_Cl and SC-9A5 were qualitatively rather than just quantitatively distinct, and the inhibitions of glycoprotein conformations (Fig. 3, Fig. 4) correlated with the inhibition of tegument protein release (Fig. 5).

### Pre-fusion antigenic changes in gp150

**Figure 6:**
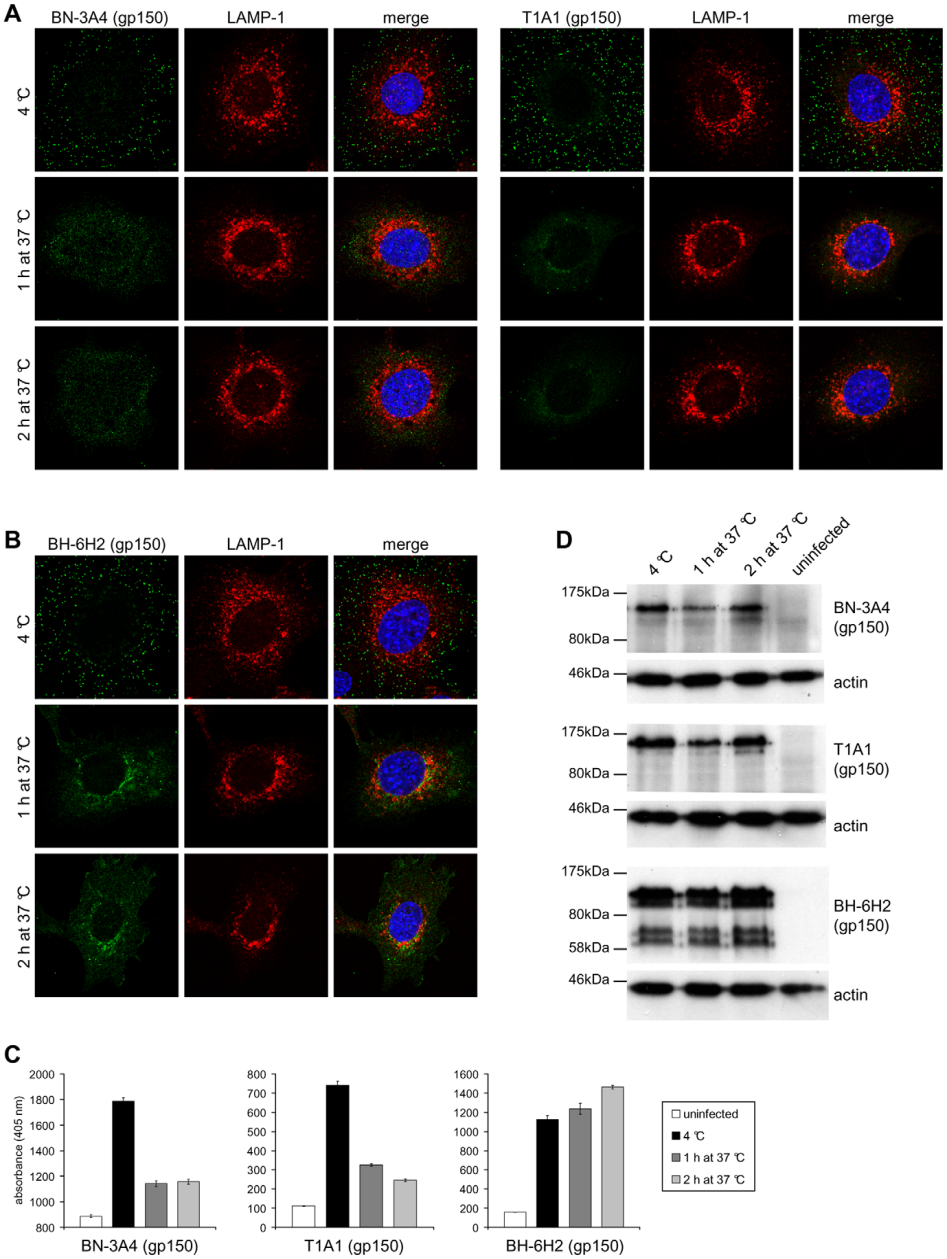
Antigenic changes in gp150 during virus entry. **(A)** NMuMG cells were incubated with MuHV-4 (3 p.f.u./cell, 2h, 4°C), washed, and then either fixed immediately or first further incubated (1h and 2h, 37°C) to allow virion endocytosis. The cells were then stained with the gp150-specific mAbs BN-3A4 and T1A1 (green). BN-3A4 (IgG_1_) recognizes an epitope in the gp150 N-terminal (amino acid residues 1-150), T1A1 (IgG_2a_) an epitope in the central (residues 150-250). **(B)** Cells were infected and processed as in **(A)** but stained with mAb BH-6H2 (IgG_2b_), which recognises an epitope in the gp150 C-terminal (residues 301-450) extracellular domain. **(C)** Cells were infected and processed as in **(A)**. Infected cells and uninfected control cells were then incubated with the gp150-specific mAbs BN-3A4 (IgG_1_), T1A1 (IgG_2a_), and BH-6H2 (IgG_2b_). Bound antibody was detected with alkaline phosphatase-conjugated secondary antibodies and incubation with p-nitrophenyl phosphate substrate. The bars show mean ± SEM values from 6 wells. The experiment shown is representative of two equivalent experiments. **(D)** Cells were infected and processed as in **(A)**. Lysates of infected cells and uninfected control cells were then analyzed by immunoblot with the gp150-specific mAbs BN-3A4 (IgG_1_), T1A1 (IgG_2a_), and BH-6H2 (IgG_2b_). Detection of actin served as loading control.

Since gp150 is also part of the MuHV-4 entry complex [23], we looked to see whether it too showed entry-associated antigenic changes. We used mAbs that recognize epitopes in the N-terminal (amino acid residues 1-150, mAb BN-3A4), central (residues 150-250, mAb T1A1) or C-terminal (residues 301-450, mAb BH-6H2) regions of the gp150 extracellular domain. All these mAbs recognized linear epitopes (Fig. S5). BN-3A4 recognition was further mapped by ELISA to a 15-mer peptide ETEPESPTPLPATPK, corresponding to gp150 residues 127-141 (data not shown). As before we bound virions to cells at 4°C, and then either fixed and stained them immediately or first incubated them at 37°C to allow endocytosis. Each mAb recognized virions bound to the plasma membrane (Fig. 6A, 6B). After 1h at 37°C, recognition by BN-3A4 and T1A1 (Fig. 6A) was greatly reduced while recognition by BH-6H2 (Fig. 6B) was retained. Some redistribution of BH-6H2 staining was evident, particularly after 2h at 37°C.

**Figure 7:**
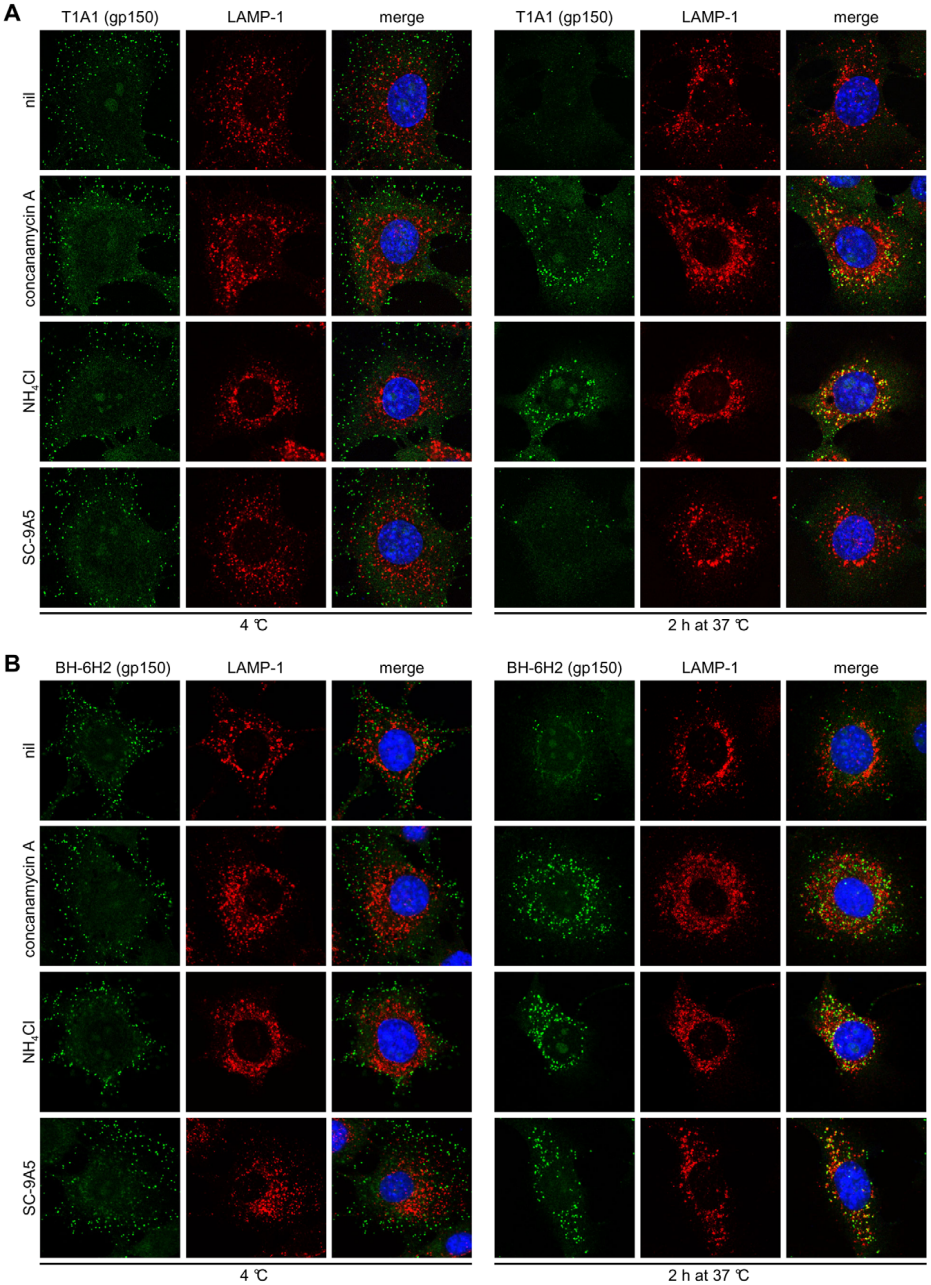
Gp150 undergoes pre-fusion antigenic changes. Infections, drug treatments and antibody treatments were as for Figure 3. In **(A)** the cells were stained with the gp150-specific IgG_2a_ BN-3A4, in **(B)** with the gp150-specific IgG_2a_ T1A1 (green), and in **(C)** with the gp150-specific IgG_2b_ BH-6H2. The cells were also stained for LAMP-1 (red) and with DAPI (blue). Treating cells with DMSO alone had no effect (not shown).

Signal quantitation by ELISA (Fig. 6C) confirmed these results. Although the loss of staining by BN-3A4 and T1A1 was a loss of linear epitope recognition, immunoblots (Fig. 6D) showed no sign of gp150 degradation. Therefore 2 epitopes in distinct regions of the gp150 extracellular domain became hidden by rearrangement/conformation changes of the virion fusion complex. ConA and NH_4_Cl preserved gp150 recognition by T1A1 (Fig. 7A) and BN-3A4 (Fig. S6). Therefore these changes were pH-dependent. MAb SC-9A5, which blocked membrane fusion, did not prevent T1A1 and BN-3A4 epitope loss. Therefore these changes were also pre-fusion. In contrast all 3 treatments blocked the redistribution of BH-6H2 staining (Fig. 7B). Therefore this seemed to reflect membrane fusion.

### The fusion complex changes are not simply pH-dependent

**Figure 8:**
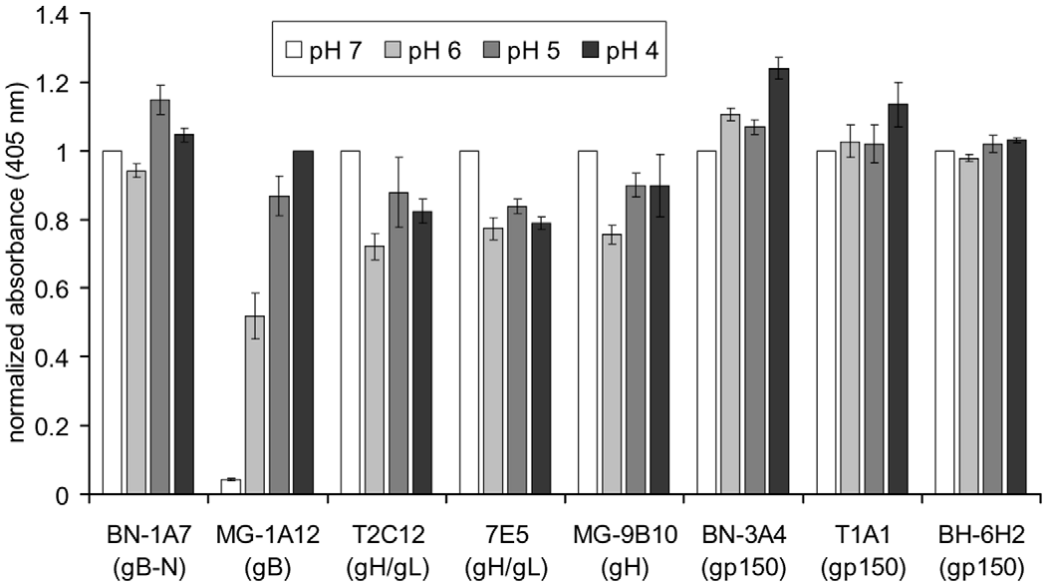
Exposing virions to low pH reveals the gB MG-1A12 epitope, but fails to trigger antigenic changes in gH and gp150. Virions (10^5^ p.f.u./well) were absorbed to 96-well ELISA plates at neutral pH (overnight, 4°C). After washing to remove unbound virions, the plates were treated with phosphate-citrate buffers with pH 7, 6, 5, and 4 (15 min, 37°C). Virions were then probed with gB-specific mAbs BN-1A7 (IgG_2a_) and MG-1A12 (IgG_2a_), gH/gL-specific mAbs T2C12 (IgG_2a_) and 7E5 (IgG_2a_), the gH-only-specific mAb MG-9B10 (IgG_2a_), and gp150-specific mAbs BN-3A4 (IgG_1_), T1A1 (IgG_2a_) and BH-6H2 (IgG_2b_). Bound antibody was detected with an alkaline phosphatase-conjugated secondary antibody and incubation with p-nitrophenyl phosphate substrate. For each individual mAb, the absorbance values were normalized to the value obtained at pH 7, except for mAb MG-1A12, where the absorbance values were normalized to the value obtained at pH 4. The bars show mean ± SEM values from 3 experiments.

Low pH is a well-established trigger of glycoprotein conformation changes for endocytic viruses [10], and the antigenic changes in MuHV-4 glycoproteins required low endosomal pH. However, when virions were bound to ELISA plates and exposed to low pH here rather than in endosomes (Fig. 8), the only marked effect was to increase MG-1A12 recognition. This was surprising, as while MG-1A12 epitope display was blocked by ConA treatment, it was not blocked by NH_4_Cl. The likely explanation is that MG-1A12 epitope display requires only a small pH drop - 10mM NH_4_Cl would still allow the endosomal pH to reach 6.5 [31]. This would be consistent with its appearance in early endosomes (Fig. 1C). That low pH alone was insufficient to drive the other MuHV-4 antigenic changes implied that receptor binding and other aspects of the endosomal milieu might also play important roles.

### Independence and inter-dependence in the changes to gH and gp150

**Figure 9:**
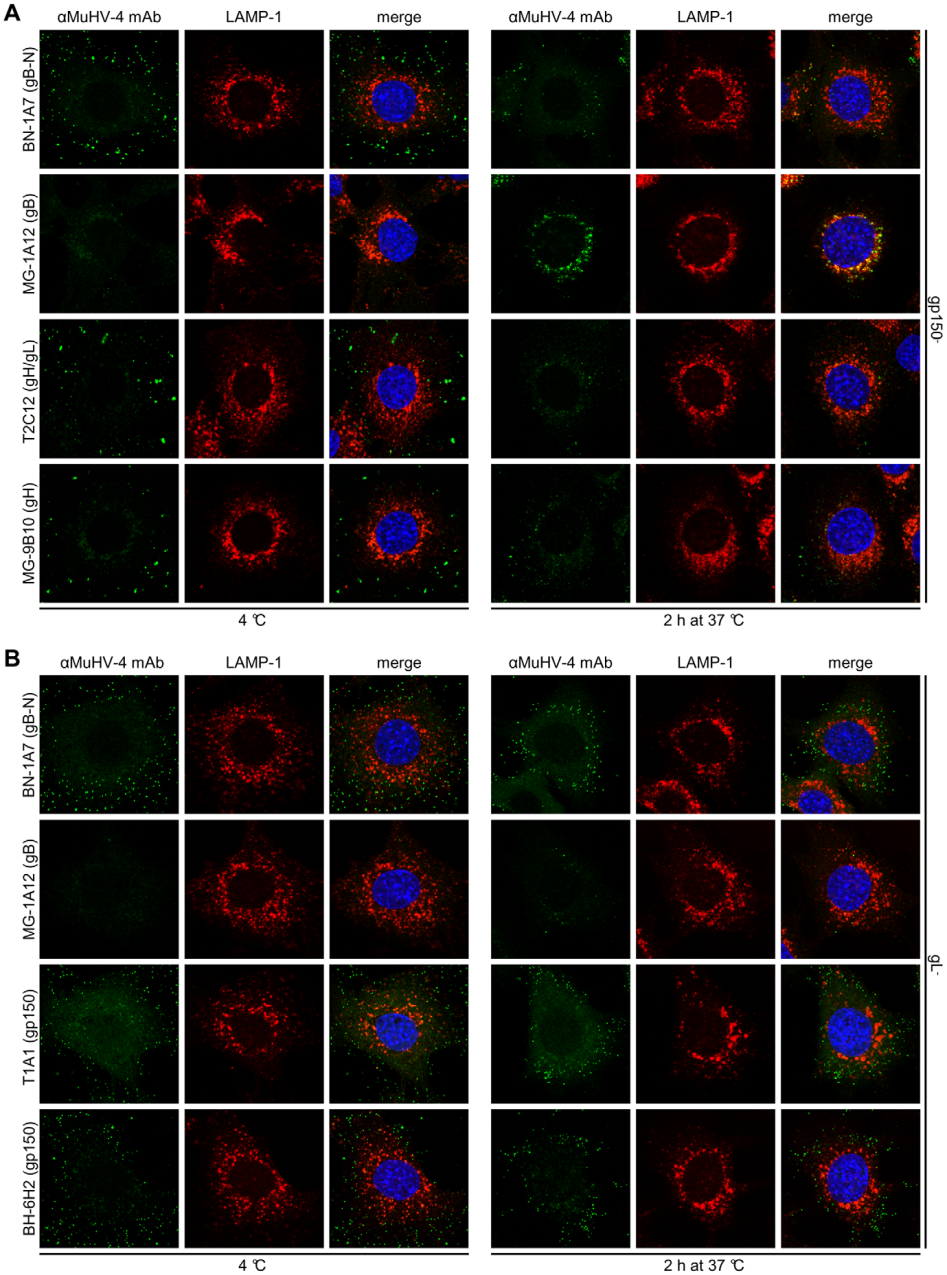
The pre-fusion antigenic changes in gB, gH, and gp150 occur independently of each other. **(A)** NMuMG cells were incubated with gp150^-^ MuHV-4 (2h, 4°C), washed, and then either fixed immediately or first further incubated (2h, 37°C) to allow virion endocytosis. Cells were then stained with the gB-specific mAbs BN-1A7 (IgG_2a_) and MG-1A12 (IgG_2a_), the gH/gL-specific mAb T2C12 (IgG_2a_), and the gH-only-specific mAb MG-9B10 (IgG_2a_) (green). The cells were also stained for LAMP-1 (red) and with DAPI (blue). gp150^-^ virus showed the same gB and gH changes as wild-type. **(B)** NMuMG cells were incubated with gL^-^ MuHV-4 (2h, 4°C), washed, and then either fixed immediately or first further incubated (2h, 37°C) to allow virion endocytosis. Cells were then stained with the gB-specific mAbs BN-1A7 (IgG_2a_) and MG-1A12 (IgG_2a_) and the gp150-specific mAbs T1A1 (IgG_2a_) and BH-6H2 (IgG_2b_) (green). The cells were also stained for LAMP-1 (red) and with DAPI (blue). gL^-^ virions bound to the cell surface displayed the same gB and gp150 epitopes as wild-type, but were only poorly transported to LAMP-1^+^ late endosomes upon incubation at 37°C. Consequently, gB and gp150 largely remained in their extracellular form.

The correlation across different inhibitor treatments of ORF75c release, BN-1A7^+^ gB loss, and MG-9B10^+^ gH and BH-6H2^+^ gp150 redistributions, argued that these are all aspects of a common fusion event. In contrast, the pre-fusion changes in gH and gB - gH/gL epitope loss and MG-1A12 epitope gain - showed at least partial independence: NH_4_Cl treatment established that gH/gL loss was unnecessary for MG-1A12 gain, and that MG-1A12 gain was insufficient for gH/gL loss. We used knockout viruses to look for inter-dependence of the pre-fusion changes in gH/gL and gp150 (Fig. 9). Again we bound virions to cells at 4°C then incubated at 37°C to allow endocytosis, but compared wild-type virions with those lacking either gp150 (BN-3A4^-^T1A1^-^BH-6H2^-^) or gL (gH/gL^-^gH-only^+^). Gp150^-^ virions showed the same gH and gB changes as wild-type (Fig. 9A). Therefore gp150 did not hide the MG-1A12 epitope or obviously affect gH/gL. gL^-^ virions also displayed each gp150 epitope, so gH/gL dissociation was not responsible for gp150 epitope loss (or for MG-1A12 epitope gain). gL^-^ virions are poorly transported to late endosomes [22]. Thus even after 2h at 37°C they remained largely BN-1A7^+^MG-1A12^-^ (no pre-fusion change in gB) and T1A1^+^ (no pre-fusion change in gp150) (Fig. 9B). They also remained BN-3A4^+^ (data not shown). Therefore bar a common requirement for virion endocytosis and low pH, the pre-fusion changes in gH, gB and gp150 showed substantial independence.

## Discussion

MuHV-4 virions show multiple glycoprotein antigenic changes as they enter epithelial cells. Inhibitors of membrane fusion identified gB MG-1A12 epitope gain, gp150 BN-3A4 and T1A1 epitope loss and gH/gL epitope loss as pre-fusion events. Each required low pH, but low pH alone triggered only the change in gB. Therefore other aspects of the endosomal milieu were required to change gH/gL and gp150. All these changes depended on virion endocytosis, and the antigenic switching between virions at the cell surface and those engaging in fusion in late endosomes presumably helps MuHV-4 to evade antibody-mediated neutralization.

An important question is what structural/organizational changes in the virion fusion complex the antigenic changes represent. The MG-1A12 epitope on gB was not hidden by gH/gL or gp150, as it was revealed before their antigenicity changed, and was not revealed when virions lacked gp150 or gL. It presumably reflected instead a change in gB itself. Another gB antigenic change - BN-1A7 epitope loss - then occurred upon fusion. Thus gB could undergo sequential conformation changes (BN-1A7^+^MG-1A12^-^ to BN-1A7^+^MG-1A12^+^ to BN-1A7^-^MG-1A12^+^), or the switch to BN-1A7^+^MG-1A12^+^ could reflect a reversible equilibrium being established between BN-1A7^+^MG-1A12^-^ (pre-fusion) and BN-1A7^-^MG-1A12^+^ (post-fusion) gB, as described for VSV-G [10]. An irreversible switch to post-fusion would then be driven by membrane binding [29]. Existing structural data cannot resolve these possibilities, as only post-fusion gB has been identified. Low pH induces local changes in the post-fusion HSV gB [13], but this has uncertain functional significance as HSV fusion is classically pH-independent [32]; different changes may occur in other recombinant forms of HSV gB [33] but remain structurally uncharacterized.

Fusion-associated changes were less clear for gH and gp150. This was unsurprising for gp150, as it probably does not participate directly in fusion. However, fusion does require gH. The MuHV-4 gH can be either gH/gL or gH-only. gL is non-essential for infectivity, and virions reaching their fusion site (late endosomes) lost gH/gL epitopes even when mAb SC-9A5 blocked fusion. Therefore fusion involves gH-only rather than gH/gL, and the gH/gL of extracellular virions [23] must change to gH-only before fusion occurs. That other herpesviruses require gL to process gH [34] and incorporate it into virions [35], [36] does not exclude that they follow a similar scheme.

The MuHV-4 gH/gL binds to HS [18]; it may also deliver a pro-endocytic signal, as a major phenotype of gL^-^ virions is poor transport to late endosomes. Late endosomal gH/gL loss must therefore reduce cell binding. Gp150 regulates HS-independent cell binding, and the late endosomal loss of BN-3A4 and T1A1 epitopes could have reflected gp150 displacement for a compensatory increase in HS-independent cell binding. If gH-only engages directly in membrane fusion, another gH conformation change might be expected, as with gB (BN-1A7 epitope loss), and the immunofluorescence data did suggest that gH-only epitopes are lost when fusion occurs. However the ELISA data argued that fusion mainly dilutes gH-only by allowing its redistribution. Therefore it remains possible that gH-only simply licences fusion by gB.

We know that gL protects MuHV-4 against neutralization by gH-only-specific antibodies [37], and that BN-1A7^+^MG-1A12^+^ gB was inaccessible on extracellular virions could explain the difficulty [19] of gB-directed MuHV-4 neutralization. MAb SC-9A5 neutralizes well, but its epitope is rarely recognized by MuHV-4-infected or gB-vaccinated mice [38], suggesting that it is hard to reach. Gp150 seems not to be a significant neutralization target [39]. Thus the post-endocytic reorganization of gH, gB and gp150 makes sense as a mechanism of viral antibody evasion.

## Materials and Methods

### Cells

NMuMG epithelial cells (American Type Culture Collection CRL-1636), BHK-21 fibroblasts (CCL-10), and 293T cells (CRL-11268) were grown in Dulbecco's Modified Eagle's Medium with 2 mM glutamine, 100 U/ml penicillin, 100 µg/ml streptomycin and 10% fetal calf serum (complete medium).

### Plasmids

N-terminal fragments (amino acid residues 1-150, 1-250 and 1-450) of the gp150 coding sequence (genomic co-ordinates 69466-70917) [40] were amplified by PCR (Hi-Fidelity PCR kit, Roche Diagnostics Ltd) using 5′ *Xba*I-restricted and 3′ *Not*I-restricted primers, and cloned into the *Xba*I/*Not*I sites of pBRAD, thereby attaching a C-terminal glycosyl-phosphatidyl-inositol (GPI) membrane anchor [41]. The 301-450 amino acid residue fragment was amplified instead with a *Sma*I-restricted 5′ primer, then cloned as a *Sma*I/*Not*I fragment into the equivalent sites of pBRAD-gp150 (1-150). This removed gp150 residues 31-150 from the original plasmid and joined residue 29 to residue 301 via a *Sma*I restriction site. The predicted gp150 signal sequence cleavage site lies between residues 22 and 23. Thus this construct retained the 7 residues after the signal sequence cleavage site then encoded residues 301-450. Each construct was transfected into 293T cells using Fugene-6 (Roche Diagnostics Ltd).

### Viruses

All viruses were derived from a MuHV-4 BAC [42]. gL^-^ [24] and gp150^-^ mutants [25] have been described. The loxP-flanked BAC cassette was removed from viral genomes by passage through NIH-3T3-CRE cells [25]. Virus stocks were grown in BHK-21 cells [25]. Cell debris was removed by low speed centrifugation (1000 x *g*, 10 min) and virions were recovered from supernatants by high speed centrifugation (38,000 x *g*, 90 min). Virus stocks were titrated by plaque assay [25]. After incubation with virus (2h, 37°C), BHK-21 cell monolayers were overlaid with 0.3% carboxymethylcellulose and 4 days later fixed with 4% formaldehyde and stained with 0.1% toluidine blue.

### Antibodies

MuHV-4-specific mAbs were derived from MuHV-4-infected BALB/c mice. All animal experiments were approved by the Cambridge University ethical review board and by the UK Home Office (PPL 80/1992). Staining was with hybridoma supernatants. For virus neutralization with mAb SC-9A5, hybridoma supernatant was concentrated by ammonium sulfate precipitation, dialysed against PBS, and quantitated by Mancini assay [43]. Rat anti-mouse LAMP-1 mAb (clone 1D4B) was from BD Biosciences, rabbit anti-EEA-1 polyclonal antibody (pAb) from Abcam, and mouse anti-actin mAb (clone AC-40) from Sigma-Aldrich.

### Drug treatments

Concanamycin A (Sigma-Aldrich) stock solutions were prepared at 150 µM in DMSO. Treatment with identical volumes of DMSO served as a negative control. Ammonium chloride stock solution was prepared at 2M in H_2_O. Cells were treated for 2h at 37°C prior to addition of virus, during virus binding at 4°C, and during virus endocytosis at 37°C.

### Immunofluorescence

NMuMG cells were seeded overnight onto glass cover-slips. MuHV-4 virions (3 p.f.u./cell) were bound to the cells (2h, 4°C). The cells were then washed x3 in ice-cold PBS to remove unbound virions, and either fixed or first incubated for the indicated time at 37°C in complete medium with or without drugs and antibodies. After one wash in ice-cold PBS, fixation was achieved by adding ice-cold 4% formaldehyde in PBS and leaving at room temperature (RT) for 30 min (mAb BN-8C3) or 1h (all other mAbs). Fixation was stopped by adding 0.1M glycine (15 min, RT), followed by 3 washes in PBS. The cells were then permeabilized with 0.1% Triton X-100 (30 min, RT), blocked with 2% bovine serum albumin 0.1% Tween-20 (overnight, 4°C), stained with primary mAbs (1h, RT), washed x3 in PBS 0.1% Tween-20, stained with secondary antibodies with 1 µg/ml DAPI (1h, RT), washed x3 in PBS 0.1% Tween-20 and x1 in H_2_O, and mounted in ProLong Gold (Invitrogen). Secondary antibodies (goat anti-rat IgG, goat anti-rabbit IgG, and goat anti-mouse IgG, IgG_1_, IgG_2a_, IgG_2b_ or IgG_3_, labeled with Alexa Fluor 488 or 568) were all from Invitrogen. Images were acquired on Leica TCS SP2 and SP5 AOBS confocal laser scanning microscopes with settings specific for DAPI (excitation 405 nm, recording 410–470 nm), Alexa Fluor 488 (excitation 488 nm, recording 493-550 nm), and Alexa Fluor 568 (excitation 561 nm, recording 566–700 nm). Images were analyzed with ImageJ.

### Immunoblotting

Cells or virions were lysed and denatured by heating in Laemmli's buffer (95°C, 5 min for virions and 15 min for cells). Proteins were resolved by SDS-PAGE and transferred to polyvinylidene difluoride (PVDF) membranes (Millipore). The membranes were blocked for 1h at RT with 5% skim milk in PBS-T (PBS + 0.3% Tween-20) and probed with MuHV-4-specific mAbs diluted in 2.5% skim milk in PBS-T (1h, RT), followed by 4 washes in PBS-T. The membranes were then incubated with horseradish peroxidase-conjugated rabbit anti-mouse Ig pAb (DakoCytomation) diluted in 2.5% skimmed milk in PBS-T (1 h, RT), followed by 4 washes in PBS-T and 1 wash in PBS. Bound secondary antibody was detected with Amersham ECL Western blotting detection reagent (GE Healthcare), followed by exposure to X-ray films (Fuji).

### Flow cytometry

Transfected or MuHV-4-infected cells were trypsinized and washed in PBS. Cells were then incubated (1 h, 4°C) with MuHV-4 glycoprotein-specific mAbs followed by fluorescein-conjugated rabbit anti-mouse IgG pAb (Dako Cytomation) diluted in 5% rabbit serum. All samples were washed x2 in PBS and analyzed on a FACSCalibur (BD Biosciences).

### Cell ELISA

NMuMG cells were seeded overnight into 96-well plates. MuHV-4 virions (3 p.f.u./cell) were bound to the cells (2h, 4°C). The cells were then washed x3 in ice-cold PBS to remove unbound virions, and either fixed directly or first incubated at 37°C in complete medium. After one wash in ice-cold PBS, cells were fixed by adding ice-cold 4% formaldehyde in PBS and leaving at RT for 1h. Fixation was then stopped by incubation with 0.1 M glycine (15 min, RT), followed by 3 washes in PBS. The cells were then permeabilized with 0.1% Triton X-100 (30 min, RT), blocked with 2% bovine serum albumin 0.1% Tween-20 (overnight, 4°C), then incubated with primary mAbs diluted in 2% bovine serum albumin 0.1% Tween-20 (2h, RT), followed by 3 washes in PBS 0.1% Tween-20. The plates were then incubated with alkaline phosphatase-conjugated goat anti-mouse IgG1, IgG2a, and IgG2b pAb (SouthernBiotech) diluted in 2% bovine serum albumin 0.1% Tween-20 (2h, RT), followed by 6 washes in PBS 0.1% Tween-20. Bound secondary antibodies were detected by incubation with SIGMA FAST p-nitrophenyl phosphate substrate (Sigma-Aldrich) and reading the absorbance at 405nm wavelength on a Benchmark Microplate Reader (BioRad).

### Virion ELISA

MuHV-4 virions (10^5^ p.f.u./well) were absorbed to 96-well MaxiSorp Nunc-Immuno Plates (Thermo Scientific) in PBS pH 7.4 (overnight, 4°C). After 3 washes in PBS, virions were treated with phosphate-citrate buffers pH 7, 6, 5, and 4 (15 min, 37°C). The virions were then fixed by adding ice-cold 4% formaldehyde in PBS and leaving at RT for 1h. Fixation was stopped by treatment with 0.1 M glycine (15 min, RT), followed by 3 washes in PBS. The plates were then treated with 0.1% Triton X-100 (30 min, RT), blocked with 3% bovine serum albumin (1h, RT), then incubated with primary mAbs diluted in 3% bovine serum albumin (1h, RT), followed by 3 washes in PBS. The plates were then incubated with alkaline phosphatase-conjugated goat anti-mouse IgG pAb (Sigma-Aldrich) diluted in 5% normal goat serum (1h, RT), followed by 5 washes in PBS. Bound secondary antibodies were detected by incubation with SIGMA FAST p-nitrophenyl phosphate substrate (Sigma-Aldrich) and reading the absorbance at 405 nm wavelength on a Benchmark Microplate Reader (BioRad).

## Supporting Information

Figure S1 **Low magnification images of antigenic changes in gB and gH. (A)** NMuMG cells were left uninfected or incubated with MuHV-4 (3 p.f.u./cell, 2h, 4°C), washed, and then either fixed immediately or first further incubated (1h and 2h, 37°C) to allow virion endocytosis. The cells were then stained with the gB-specific mAbs BN-1A7 (IgG_2a_) and MG-1A12 (IgG_2a_), the gH/gL-specific mAb T2C12 (IgG_2a_), and the gH-only-specific mAb MG-9B10 (IgG_2a_) (green). The cells were counter-stained with DAPI (blue).
(PDF)

Figure S2 **Kinetic analysis of gH recognition by the gH/gL-specific mAb 7E5. (A)** NMuMG cells were incubated with MuHV-4 (3 p.f.u./cell, 2h, 4°C), washed, and then either fixed immediately or first further incubated (1h and 2h, 37°C) to allow virion endocytosis. The cells were stained with the gH/gL-specific IgG_2a_ 7E5 (green), a LAMP-1-specific mAb (red), and DAPI (blue). **(B)** Cells were infected and processed as in **(A)**. Infected cells and uninfected control cells were then incubated with the gH/gL-specific mAb 7E5 (IgG_2a_) and bound antibody detected with an alkaline phosphatase-conjugated secondary antibody and incubation with p-nitrophenyl phosphate substrate. The bars show mean ± SEM values from 6 wells. The experiment shown is representative of two equivalent experiments. **(C)** Cells were infected and processed as in **(A)**, followed by staining with the gH/gL-specific IgG_2a_ 7E5 (green) and DAPI (blue).
(PDF)

Figure S3 **Concanamycin A and NH_4_Cl treatments and virus neutralization by SC-9A5 have no effect on recognition by a pan-gB-specific mAb.** Infections, drug treatments and antibody treatments were as for Figure 3. The cells were stained with the pan-gB-specific IgG_2a_ MG-4D11 (green), a LAMP-1-specific mAb (red), and DAPI (blue).
(PDF)

Figure S4 **NH_4_Cl treatment and virus neutralization by SC-9A5 lead to retention of virions within LAMP-1^+^ endosomes for several hours.** Infections, drug treatments and antibody treatments were as for Figure 3.The cells were stained with the ORF75c-specific IgG_1_ BN-8C3 (green), a LAMP-1-specific mAb (red), and DAPI (blue).
(PDF)

Figure S5 **Characterization of gp150-specific mAbs. (A)** BHK-21 cells were infected (2 p.f.u./cell, 18h) with wild-type (WT) or gp150-deficient (M7^-^) MuHV-4 or left uninfected (UI). The cells were then stained with gp150-specific mAbs or with the gN-specific mAb 3F7 as a control, and analyzed by flow cytometry. Thus BN-3A4, T1A1 and BH-6H2 were all gp150-specific. **(B)** 293T cells were transfected with expression plasmids for glycosyl-phosphatidyl-inositol-linked forms of residues 1-150, 1-250, 1-450 or 301-450 of the full-length 463 amino acid gp150 extracellular domain. The cells were then stained with gp150-specific mAbs and analyzed by flow cytometry. Arrows indicate positive staining. MAbs BN-3A4 and T1A1 also recognized the corresponding regions of gp150 expressed as GST fusion proteins in *E. coli*. MAb BH-6H2 did not, presumably because its recognition is glycan-dependent (data not shown). **(C)** Wild-type MuHV-4 virions were denatured and immunoblotted with anti-gp150 mAbs or with mAb 3F7 (anti-gN) as a control. The faster migrating bands detected by BH-6H2 did not appear to be C-terminal gp150 cleavage products as corresponding N-terminal fragments were not detected in infected cell supernatants (data not shown). Also no spliced gp150 mRNA was detected in infected BHK-21 cells by PCR (data not shown). Thus they are likely to be alternative glycoforms in which the BN-3A4 and T1A1 epitopes are hidden.
(PDF)

Figure S6 **The epitope of mAb BN-3A4 on gp150 is lost pre-fusion.** Infections, drug treatments and antibody treatments were as for Figure 3. The cells were stained with the gp150-specific IgG_1_ BN-3A4 (green), a LAMP-1-specific mAb (red), and DAPI (blue).
(PDF)
