# Supplementary figures and images for: Herpesvirus Glycoproteins Undergo Multiple Antigenic Changes before Membrane Fusion
Source: PLoS One. 2012 Jan 9;7(1):e30152. doi: 10.1371/journal.pone.0030152 (PMC3253813; doi:10.1371/journal.pone.0030152)

Figure S1

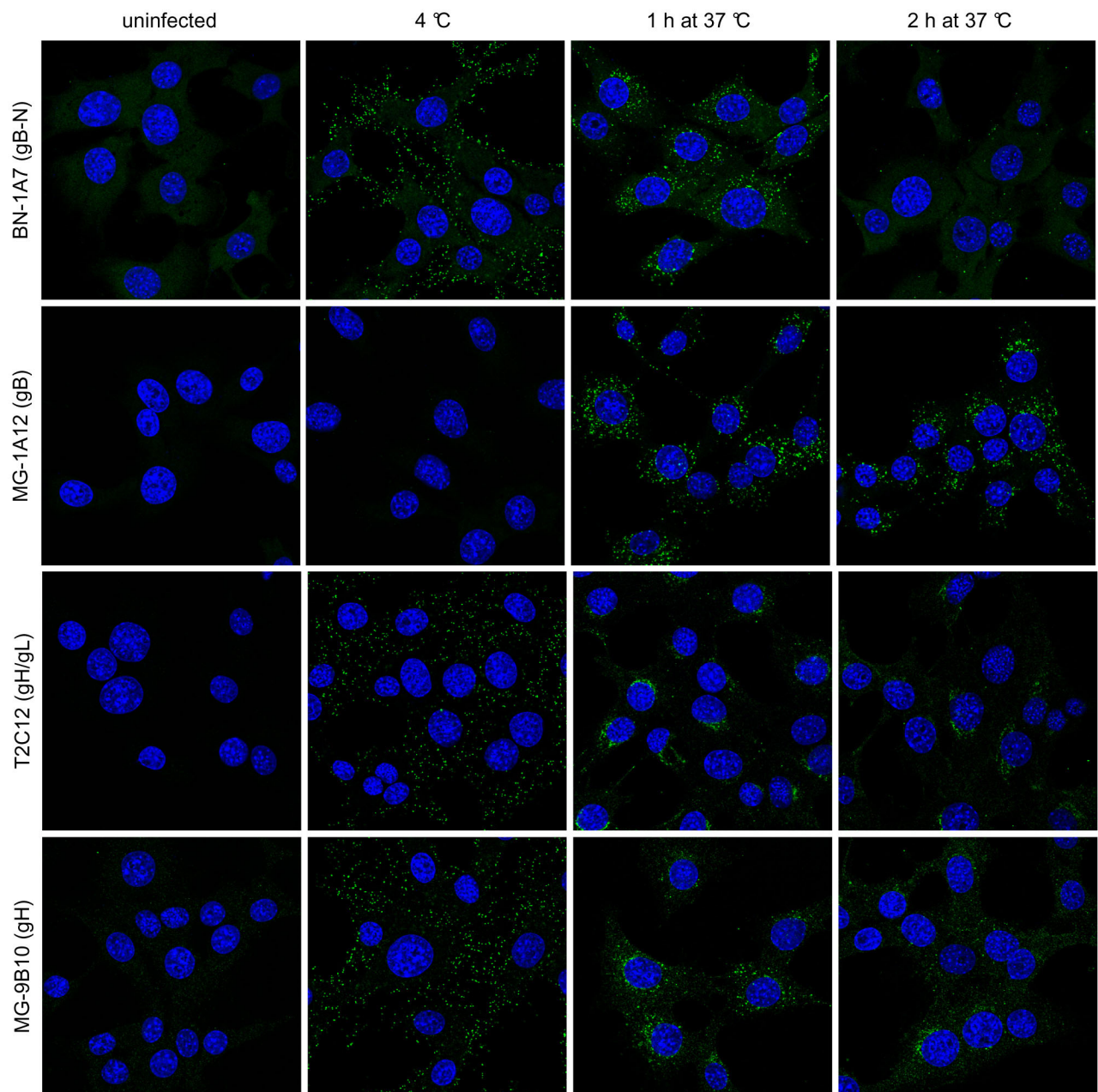

Supplement: Figure S1 — Low magnification images of antigenic changes in gB and gH. (A) NMuMG cells were left uninfected or incubated with MuHV-4 (3 p.f.u./cell, 2h, 4°C), washed, and then either fixed immediately or first further incubated (1h and 2h, 37°C) to allow virion endocytosis. The cells were then stained with the gB-specific mAbs BN-1A7 (IgG2a) and MG-1A12 (IgG2a), the gH/gL-specific mAb T2C12 (IgG2a), and the gH-only-specific mAb MG-9B10 (IgG2a) (green). The cells were counter-stained with DAPI (blue). (PDF) [file pone.0030152.s001.pdf]

Figure S2

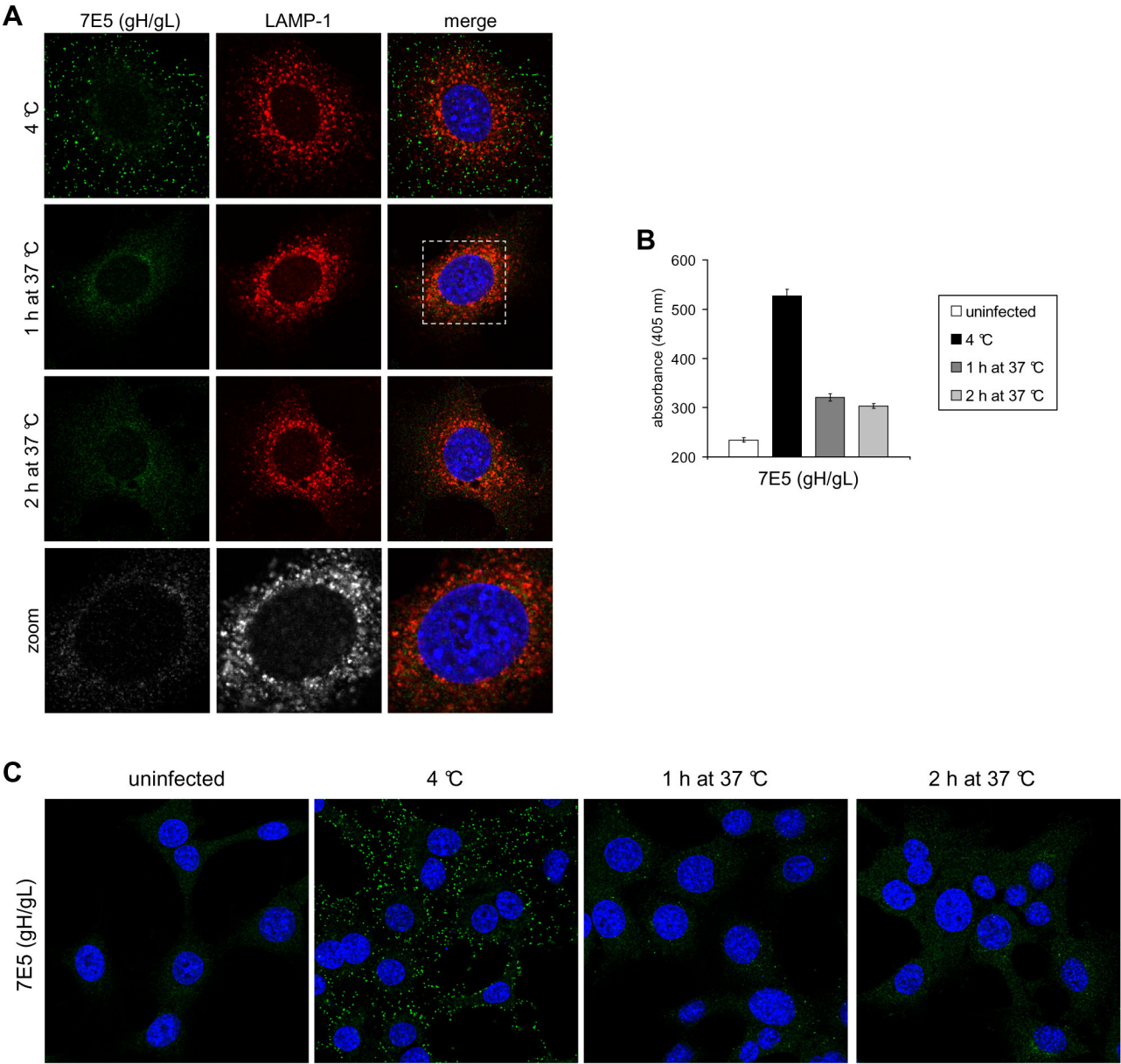

Supplement: Figure S2 — Kinetic analysis of gH recognition by the gH/gL-specific mAb 7E5. (A) NMuMG cells were incubated with MuHV-4 (3 p.f.u./cell, 2h, 4°C), washed, and then either fixed immediately or first further incubated (1h and 2h, 37°C) to allow virion endocytosis. The cells were stained with the gH/gL-specific IgG2a 7E5 (green), a LAMP-1-specific mAb (red), and DAPI (blue). (B) Cells were infected and processed as in (A). Infected cells and uninfected control cells were then incubated with the gH/gL-specific mAb 7E5 (IgG2a) and bound antibody detected with an alkaline phosphatase-conjugated secondary antibody and incubation with p-nitrophenyl phosphate substrate. The bars show mean ± SEM values from 6 wells. The experiment shown is representative of two equivalent experiments. (C) Cells were infected and processed as in (A), followed by staining with the gH/gL-specific IgG2a 7E5 (green) and DAPI (blue). (PDF) [file pone.0030152.s002.pdf]

Figure S3

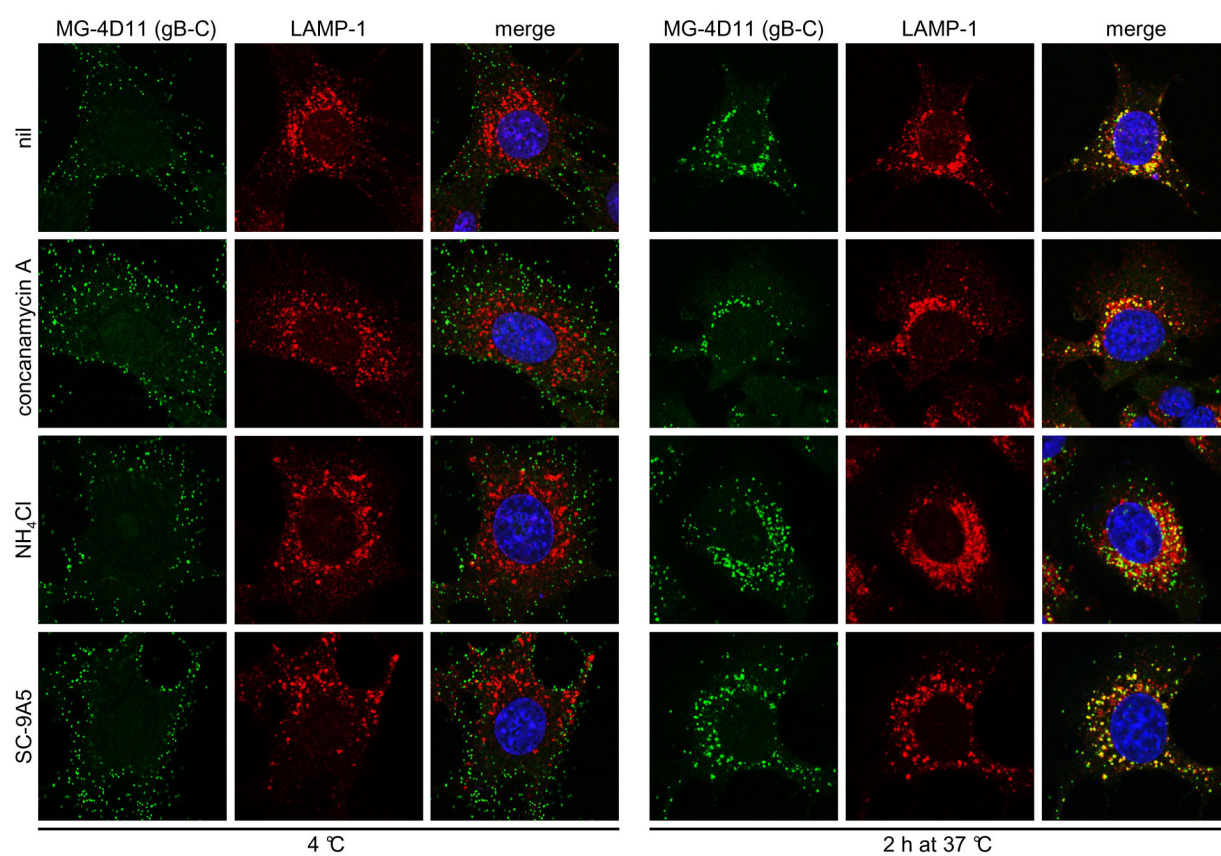

Supplement: Figure S3 — Concanamycin A and NH4Cl treatments and virus neutralization by SC-9A5 have no effect on recognition by a pan-gB-specific mAb. Infections, drug treatments and antibody treatments were as for Figure 3. The cells were stained with the pan-gB-specific IgG2a MG-4D11 (green), a LAMP-1-specific mAb (red), and DAPI (blue). (PDF) [file pone.0030152.s003.pdf]

Figure S4

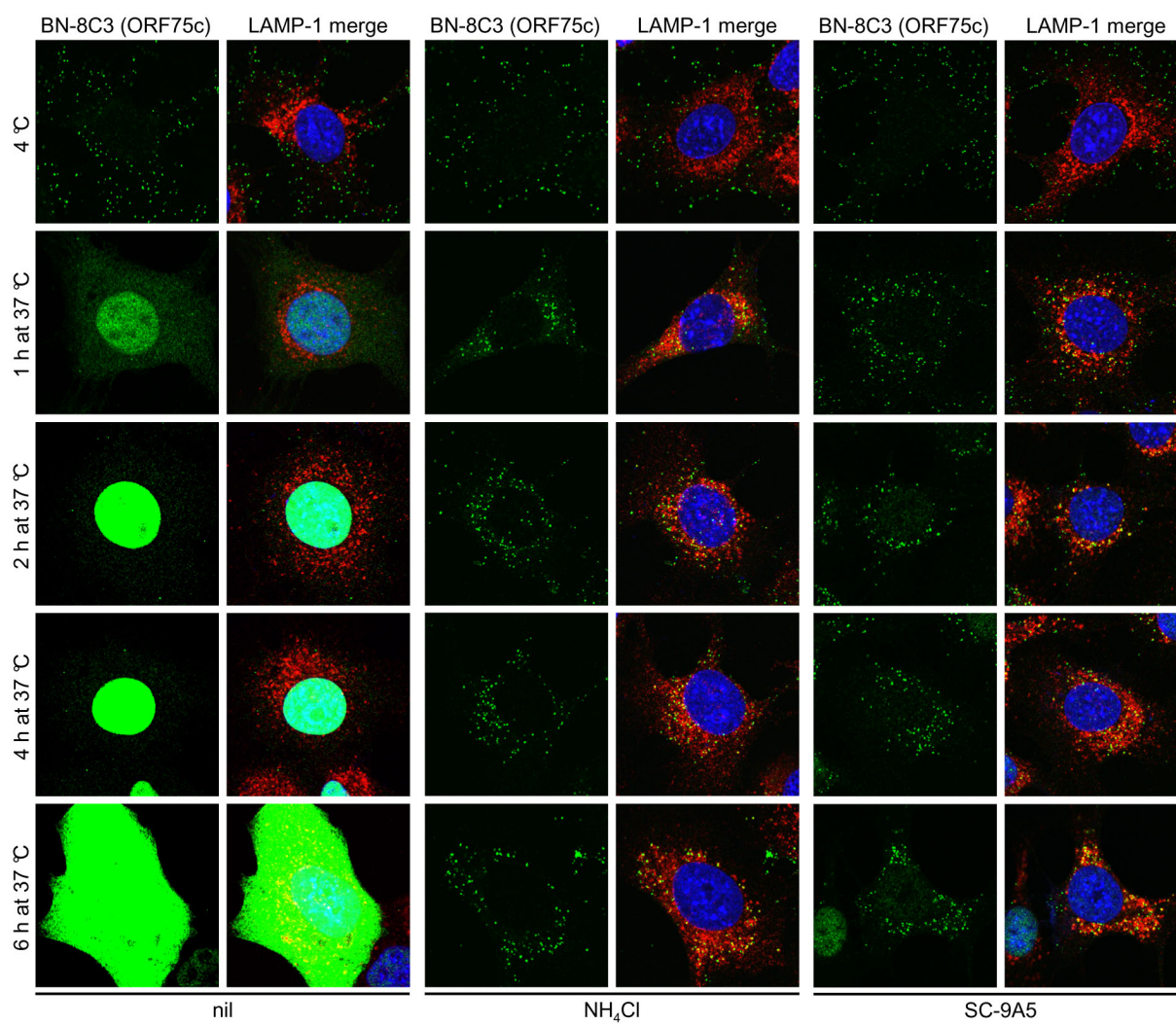

Supplement: Figure S4 — NH4Cl treatment and virus neutralization by SC-9A5 lead to retention of virions within LAMP-1+ endosomes for several hours. Infections, drug treatments and antibody treatments were as for Figure 3.The cells were stained with the ORF75c-specific IgG1 BN-8C3 (green), a LAMP-1-specific mAb (red), and DAPI (blue). (PDF) [file pone.0030152.s004.pdf]

Figure S5

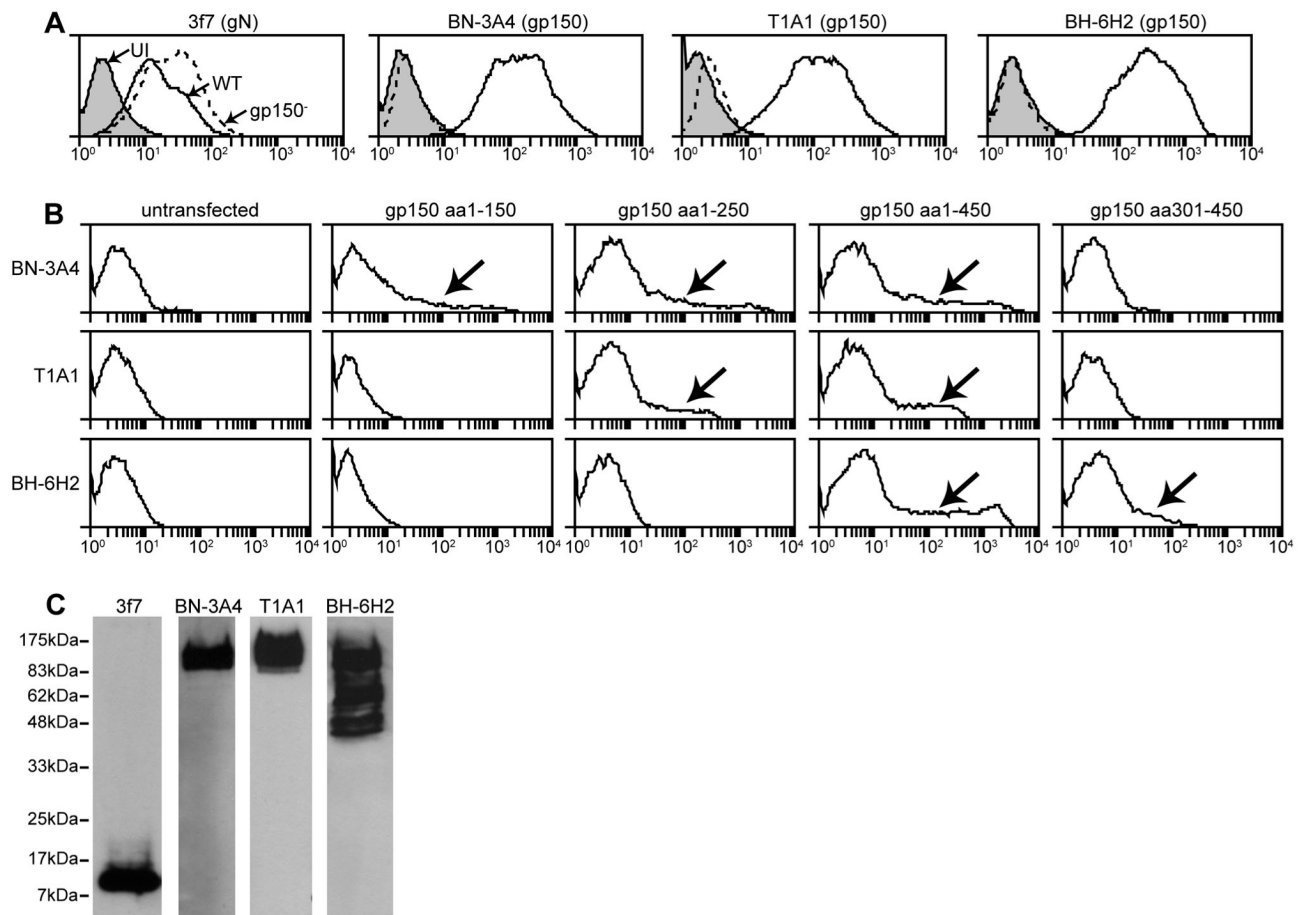

Supplement: Figure S5 — Characterization of gp150-specific mAbs. (A) BHK-21 cells were infected (2 p.f.u./cell, 18h) with wild-type (WT) or gp150-deficient (M7-) MuHV-4 or left uninfected (UI). The cells were then stained with gp150-specific mAbs or with the gN-specific mAb 3F7 as a control, and analyzed by flow cytometry. Thus BN-3A4, T1A1 and BH-6H2 were all gp150-specific. (B) 293T cells were transfected with expression plasmids for glycosyl-phosphatidyl-inositol-linked forms of residues 1-150, 1-250, 1-450 or 301-450 of the full-length 463 amino acid gp150 extracellular domain. The cells were then stained with gp150-specific mAbs and analyzed by flow cytometry. Arrows indicate positive staining. MAbs BN-3A4 and T1A1 also recognized the corresponding regions of gp150 expressed as GST fusion proteins in E. coli. MAb BH-6H2 did not, presumably because its recognition is glycan-dependent (data not shown). (C) Wild-type MuHV-4 virions were denatured and immunoblotted with anti-gp150 mAbs or with mAb 3F7 (anti-gN) as a control. The faster migrating bands detected by BH-6H2 did not appear to be C-terminal gp150 cleavage products as corresponding N-terminal fragments were not detected in infected cell supernatants (data not shown). Also no spliced gp150 mRNA was detected in infected BHK-21 cells by PCR (data not shown). Thus they are likely to be alternative glycoforms in which the BN-3A4 and T1A1 epitopes are hidden. (PDF) [file pone.0030152.s005.pdf]

Figure S6

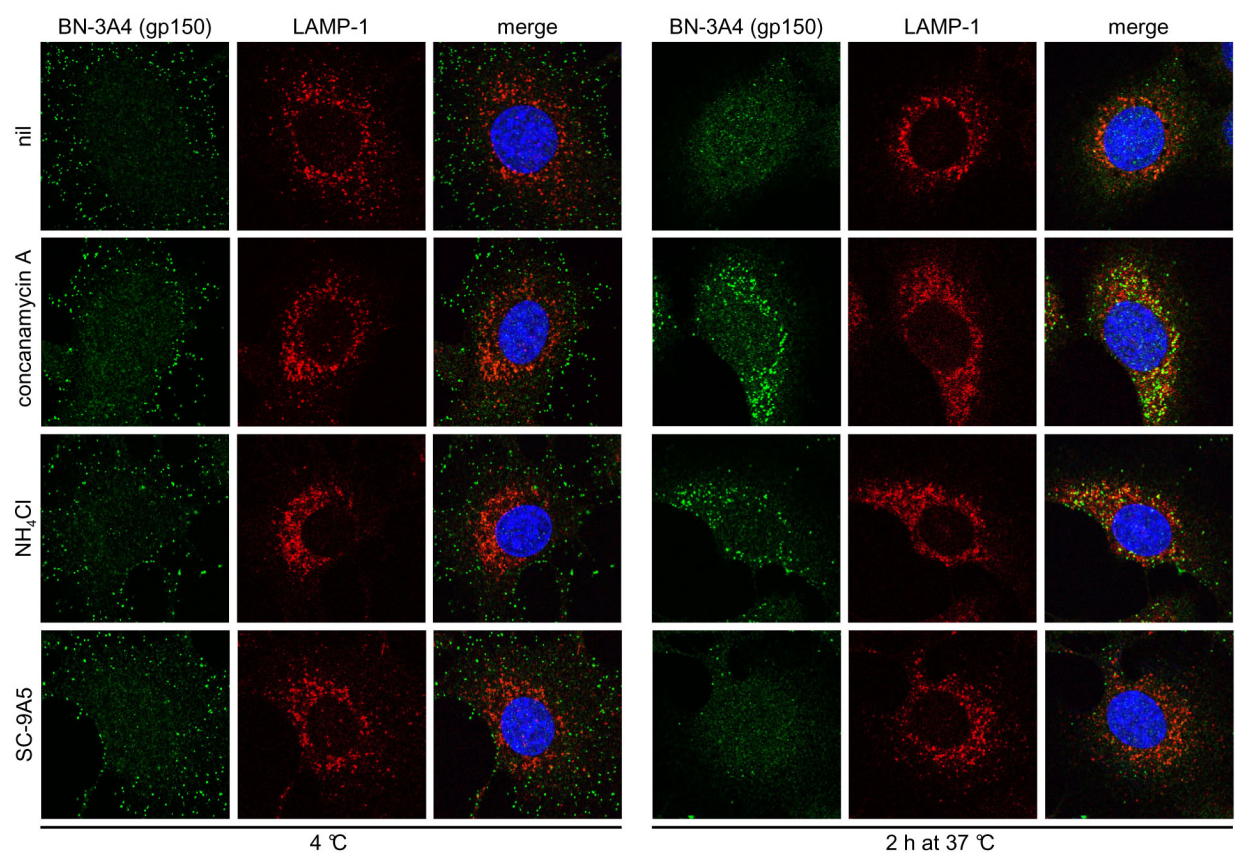

Supplement: Figure S6 — The epitope of mAb BN-3A4 on gp150 is lost pre-fusion. Infections, drug treatments and antibody treatments were as for Figure 3. The cells were stained with the gp150-specific IgG1 BN-3A4 (green), a LAMP-1-specific mAb (red), and DAPI (blue). (PDF) [file pone.0030152.s006.pdf]
